# Appropriation of mHealth Interventions for Maternal Health Care in Sub-Saharan Africa: Hermeneutic Review

**DOI:** 10.2196/22653

**Published:** 2021-10-06

**Authors:** Priscilla Maliwichi, Wallace Chigona, Karen Sowon

**Affiliations:** 1 Department of Information Systems Faculty of Commerce University of Cape Town Cape Town South Africa; 2 Department of Computer Science and Information Technology Malawi Institute of Technology Malawi University of Science and Technology Thyolo Malawi

**Keywords:** mHealth, appropriation, mobile phones, model of technology appropriation, maternal health, community of purpose, hermeneutic literature review

## Abstract

**Background:**

Many maternal clients from poorly resourced communities die from preventable pregnancy-related complications. The situation is especially grave in sub-Saharan Africa. Mobile health (mHealth) interventions have the potential to improve maternal health outcomes. mHealth interventions are used to encourage behavioral change for health care–seeking by maternal clients. However, the appropriation of such interventions among maternal health clients is not always guaranteed.

**Objective:**

This study aims to understand how maternal clients appropriate mHealth interventions and the factors that affect this appropriation.

**Methods:**

This study used a hermeneutic literature review informed by the model of technology appropriation. We used data from three mHealth case studies in sub-Saharan Africa: Mobile Technology for Community Health, MomConnect, and Chipatala Cha Pa Foni. We used the search and acquisition hermeneutic circle to identify and retrieve peer-reviewed and gray literature from the Web of Science, Google Scholar, Google, and PubMed. We selected 17 papers for analysis. We organized the findings using three levels of the appropriation process: adoption, adaptation, and integration.

**Results:**

This study found that several factors affected how maternal clients appropriated mHealth interventions. The study noted that it is paramount that mHealth designers and implementers should consider the context of mHealth interventions when designing and implementing interventions. However, the usefulness of an mHealth intervention may enhance how maternal health clients appropriate it. Furthermore, a community of purpose around the maternal client may be vital to the success of the mHealth intervention.

**Conclusions:**

The design and implementation of interventions have the potential to exacerbate inequalities within communities. To mitigate against inequalities during appropriation, it is recommended that communities of purpose be included in the design and implementation of maternal mHealth interventions.

## Introduction

### Background

Approximately 295,000 women die globally from pregnancy- and childbirth-related complications [1]. Most of these deaths are preventable [1]. The numbers are particularly high in transitional countries. For instance, sub-Saharan Africa recorded approximately 196,000 of these maternal deaths, and most of these deaths occurred in poorly resourced settings [1]. This translates to 533 deaths per 100,000 live births in sub-Saharan Africa [1]. Sustainable Development Goal 3 seeks to reduce the maternal mortality ratio to <70 deaths per 100,000 live births [1]. To contribute toward Sustainable Development Goal 3, information and communication technologies are used to improve maternal health care–seeking behavior. For example, mobile phones have been used to send health tips and reminders to visit antenatal care clinics and health facilities for delivery. The use of mobile phones in health care is known as mobile health (mHealth) [2].

Previous studies have pointed to the low uptake and low efficacy of mHealth interventions, especially in transitional countries [3,4]. For mHealth interventions to meet maternal health care needs, maternal health clients must not only adopt [5] but also appropriate interventions. Appropriation is the way technologies are adopted, adapted, and incorporated into everyday life [6]. The appropriation of technology goes beyond mere adoption. Appropriation also deals with how users engage with the technology, and this might differ from how the designers of the technology had intended. Information systems researchers have explored the phenomenon of technology appropriation [7-9]. Carroll et al [8] focused on the appropriation of technologies over time. Others have argued that the focus should be on how technologies are appropriated to get the job done or the intended outcome achieved [7]. Furthermore, technology appropriation may influence users for social changes [10]. Once technologies become a routine part of daily life, they often generate particular forms of habituated practice and a specific form of sociality.

### Objectives

There is a need to investigate the appropriation of maternal mHealth interventions by maternal clients in transitional countries [11,12]. Most studies on the appropriation of mobile technologies have been conducted in resource-rich countries where mobile phone ownership is high and infrastructure is developed [7,11]. In contrast, in transitional countries, the adoption and appropriation of mobile phones to support health care are affected by demographic factors, such as low levels of literacy and low mobile phone ownership, and structural challenges, such as low connectivity [13]. To understand mobile technology use, it is necessary to understand technology appropriation in different contexts [14]. Therefore, this study seeks to investigate how maternal health clients in transitional countries appropriate mHealth interventions. The following research questions guided this study: (1) How do maternal clients appropriate maternal mHealth interventions? (2) What factors affect the appropriation of maternal mHealth interventions?

## Methods

### Study Design

This study used a hermeneutic literature review. Data were collected and analyzed using a hermeneutic framework for reviews. This study used the model of technology appropriation (MTA) as a theoretical lens.

### Theoretical Framework: MTA

MTA was developed by Carroll et al [6] to explain how young people adopt and use technologies. MTA has been used in mHealth for decades. Imperatore and Dunlop [15] used MTA to assess how people with aphasia (lack of language abilities) appropriate smartphones. Humans interact with mobile technologies in diverse and dispersed contexts. In maternal health, maternal clients may opt to not, or fail to, exploit the capabilities of an mHealth intervention, which may result in nonappropriation of the mHealth intervention. However, deciding to register for maternal mHealth interventions initiates the process of appropriation. The process of appropriation may result in either integrating the technology in their everyday life (appropriation) or disappropriation, that is, stopping using a technology.

Technology not only shapes users’ behaviors, but users, in turn, shape how systems are created through use [9,16]. The design of systems is completed through the process of appropriation, whereby the use and performance of design change over time. Therefore, the focus of appropriation is twofold: (1) it draws attention to the context of use and the need to use evaluations that are situated in the context of the phenomenon and (2) the unfolding of use over time associated with appropriation suggests that evaluations conducted to support the design of technologies should continue after completion of the initial design process [17].

**Figure 1:**
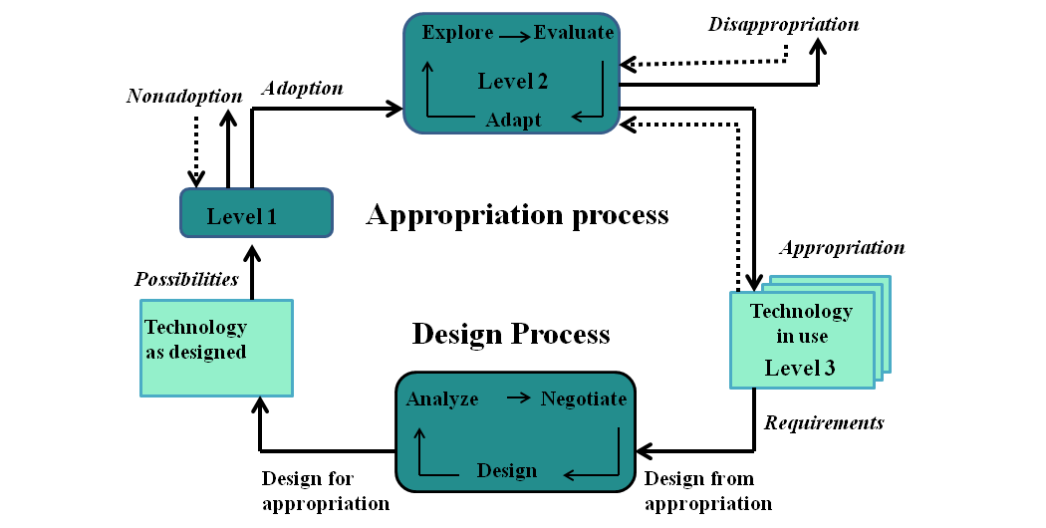
Model of technology appropriation, adapted from the study by Carroll [18].

According to MTA, the process of appropriating a technology has three stages: adoption, adaptation, and integration (Figure 1). At the *adoption stage*, the user interacts with the technology as intended by the designers [18]. Designers develop the technology to address specific needs in an organization or society. In this study, the interventions were designed to reduce maternal mortality and to assist in maternal home-based care by creating a link between the maternal client and the health facility. During the initial interaction with the technology, users evaluate the intervention and decide whether to adopt it [18]. For mHealth interventions, clients might be motivated to continue using the intervention if they find it valuable. However, a maternal client may not adopt the intervention because of other factors such as failing to register or not finding value in the use of the intervention.

At the *adaptation stage*, users evaluate the technology more by exploring and using it [18]. Users not only familiarize themselves with the technology but also learn how the technology can support their practices or needs. Carroll [18] argued that at this stage, mutual adaptation occurs, with people adapting practices associated with the use of the technology and also adapting the technology itself. During this stage, users may come across influences that can encourage or discourage them from using the technology [18]. For example, the maternal client may realize that the information they received via the intervention was helpful. However, maternal clients may *disappropriate* when the mobile phone malfunctions or encounters system failures multiple times.

At the *integration stage*, the user incorporates the technology into their everyday lives [18]. For example, a maternal client may call the intervention call center when she feels something is wrong to get advice or get referred to the clinic. At this stage, the technology is in use and is working as expected. However, maternal clients may *disappropriate* the intervention when they have a miscarriage or stillbirth.

As illustrated in Figure 1, the appropriation process was not linear. Users may move forward and backward during these stages. A user at the appropriation stage may move back to the adaptation stage or may decide to *disappropriate.* Subsequently, the user may adopt the technology again. However, over time, technologies can be evaluated and redesigned for appropriation to meet new user requirements [18]. For example, after the pilot phase, mHealth interventions can be evaluated to determine their performance. This may inform the modifications to the design of mHealth interventions.

During appropriation, users evaluate technology in use [17]. Evaluation of the performance of a product is crucial to human experience [17]; individuals evaluate the things they come across. The evaluations inform user attitudes and behaviors as well as future actions, such as recommendations to friends. These evaluations are usually informal; however, frameworks, methods, and techniques have been developed to formalize the evaluation process [17]. An example of a formal evaluation method is the mHealth Evaluation, Reporting and Assessment checklist [19].

### Hermeneutic Literature Review

#### Overview

The hermeneutic literature review was deemed appropriate for this study because of its ability to create a contextual interpretive understanding of a phenomenon under investigation. The unstructured and flexible nature of the hermeneutic literature review made a hermeneutic literature review suitable for this study [20]. The search for relevant papers when using a hermeneutic literature review extends beyond database searches, as it allows the identification of evidence through snowballing and citation tracking [21]. Furthermore, a hermeneutic literature review allows the researcher to move from a general to a more specific search to identify relevant literature [21]. This is in contrast to a systematic literature review that encourages the use of a predefined set of keywords. A systematic literature review has the limitation that it may miss publications using different wording.

When using a hermeneutics circle, understanding the meaning and importance of individual texts depends on the understanding of the whole corpus of relevant literature. In turn, an understanding of the corpus of literature is built up through the understanding of individual articles [22]. This is an iterative process. A hermeneutic literature review uses the interpretive process, whereby a researcher expands and increases their understanding of the relevant literature [22].

**Figure 2:**
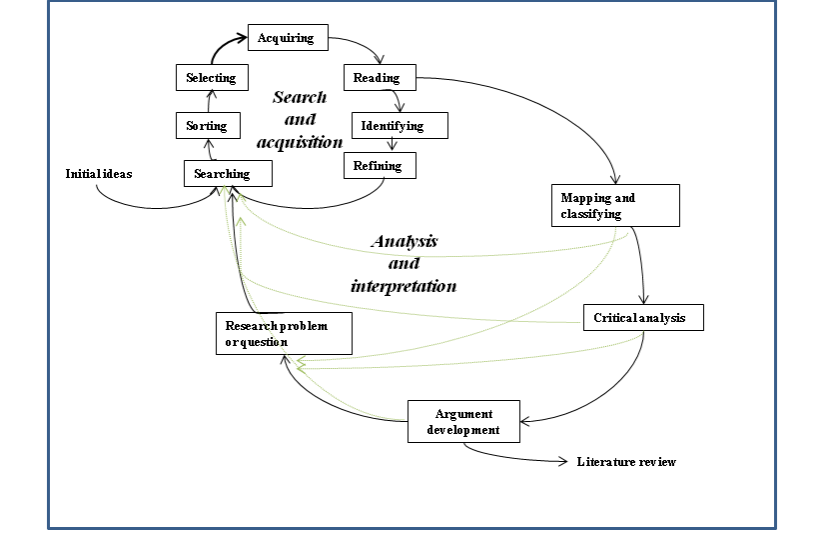
A hermeneutic framework for the literature review process [21].

Specifically, Figure 2 illustrates two circles: (1) search and acquisition and (2) analysis and interpretation. Textboxes 1 and 2 summarize the hermeneutic search and acquisition circle and the hermeneutic analysis and interpretation circle, respectively.

**Overview of the hermeneutic search and acquisition circle.**

**Activity and Description**

Searching

- When identifying publications using the hermeneutic framework, small sets of highly relevant publications are preferred over huge sets of documents whose relevance cannot be ascertained.

Sorting

- The results can be sorted based on the determined criteria, such as relevance rankings or publication dates.

Selecting

- Individual publications are selected for acquisition and reading.

Acquiring

- Full texts are acquired.

Reading

- Reading of acquired publications is initially orientational, leading to further selection of publications. Through orientational reading, the researcher gains a general understanding of the wider literature.

Identifying

- On the basis of the reading, researchers identify further search terms, additional publications (through citation tracking), authors, journals, conferences, and other sources.

Refining

- Search strategies can be used to refine searches. In particular, “citation pearl grow,” “successive fractions,” or “building blocks” can help in locating additional literature.

**Overview of the hermeneutic analysis and interpretation circle.**

**Activity and Description**

Reading

- Through analytic reading, the researcher identifies key concepts, findings, and theories and their interpretations. They also infer assumptions and a methodological approach; these may not be explicitly stated.

Mapping and classifying

- Mapping and classifying provide a systematic analysis and classification of relevant ideas, findings, and contributions to knowledge within a body of literature.

Critical assessment

- Critical assessment examines the body of literature on the basis of what is known and how knowledge is produced and acquired. The researcher also assesses how useful different types of knowledge are in understanding and explaining the problem of interest and where the boundaries and weaknesses of existing knowledge are.

Argument development

- The argument development builds from the mapping and classification and also critical assessment, leading to the construction of a gap or problematization, which provides the motivation for further research. Through argumentation, future directions of research and the rationale for specific research questions are developed.

Research problem or question

- Research questions can be formulated at a general, abstract level and at a more specific, empirical level. The former will logically follow from the gap in the literature or problematization of existing knowledge. The latter is typically transformed into one or more specific questions that can be empirically explored.

Searching

- Searching leads to the identification of additional literature for further reading.

#### Search and Acquisition Circle

Owing to the nascency of mHealth, we opted to use both peer-reviewed and gray literature to obtain holistic descriptions of mHealth interventions. We searched the Web of Science, Google Scholar, Google, and PubMed and did not impose any year restrictions. The databases were selected for their coverage of mHealth literature. We used a combination of the following search terms: *Maternal*, *mHealth*, *mobile phone*, *appropriation*, *developing countries*, *Africa*.

**Figure 3:**
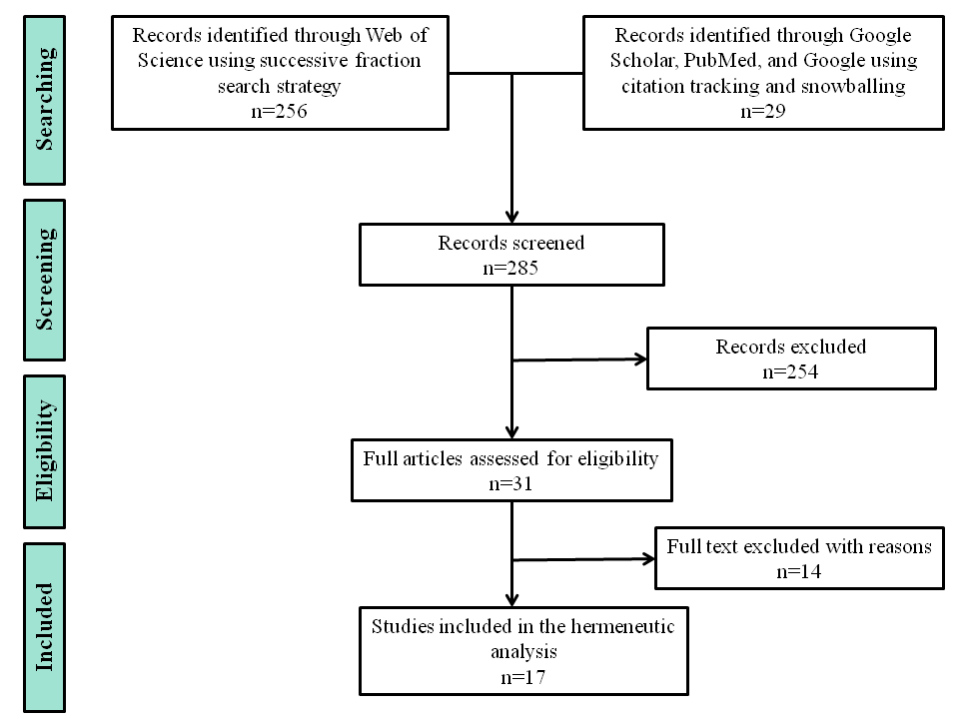
Steps involved in a hermeneutic literature review.

The details of the search and selection strategies are presented in Figure 3. The search was conducted from December 2019 to March 2020.

We read the abstracts of the identified papers. While reading, we made notes of specific ideas from the text to refine the search. This prompted a second round of search, sort, and selection, where we also used citation tracking. On the basis of this reading, we compiled a list of mHealth interventions that were implemented in sub-Saharan Africa. We were interested in the history of the interventions, the technologies used, experiences of the maternal clients, and evaluations of those technologies. Finally, we selected interventions that met the following criteria: interventions that (1) were piloted and then scaled up (this allowed us to observe the progression of the intervention), (2) had evaluated both how maternal clients used the system and the technical aspects of the intervention, (3) worked on a basic phone, and (4) had run for a minimum of 3 years.

As our unit of analysis was the maternal client, we excluded interventions where the community health workers were primary beneficiaries.

Following this search and selection process, we identified five mHealth interventions: Wired Mothers (Tanzania), Rapid SMS (Rwanda), Mobile Technology for Community Health (MOTECH; Ghana), MomConnect (South Africa), and Chipatala Cha Pa Foni (CCPF; Malawi). Only MOTECH, MomConnect, and CCPF met the inclusion criteria. Textbox 3 summarizes the interventions and publications that qualified for analysis.

**Summary of papers used for analysis in the study.**

**Project Name and Publications**

Mobile Technology for Community Health, Ghana

- Grameen Foundation [23]
- Lefevre et al [24]
- Macleod et al [25]
- Willcox et al [14]

MomConnect, South Africa

- Skinner et al [26]
- Seebregts et al [13]
- Coleman and Xiong [27]
- Lefevre et al [28]
- Barron et al [29]
- Seebregts et al [30]

Chipatala Cha Pa Foni, Malawi

- Nyemba-Mudenda and Chigona [31]
- Crawford et al [32]
- Larsen-Cooper et al [33]
- Larsen-Cooper et al [34]
- Blauvelt et al [35]
- Fotso et al [36]
- VillageReach [37]

#### Mapping and Classifying

During mapping and classifying, different factors such as the unit of analysis, major concepts, theoretical lens, and conceptual framework are considered [21]. In this study, we used MTA as the theoretical lens to map and classify our findings.

The synthesis of the selected articles involved repeated reading, looking at how different mobile technology functions have been used, and the experience of maternal clients as they appropriate these technologies for maternal health. Excel (Microsoft Inc) was used to tabulate the findings.

### Descriptions of the Interventions

#### Overview

All 3 interventions implemented mHealth interventions that could work on a basic phone. These interventions used push SMS text messaging, push voice messages, and retrieved voice messages, that is, basic functionalities of a mobile phone. A hotline service is integrated into the system to advise maternal clients in real time, and in some cases, the helpdesk is used to report queries encountered when appropriating the intervention.

All 3 interventions in this study evaluated the technological performance of their mHealth intervention after the pilot phase and after operating for a few years after scaling up. This enabled the implementer to modify the system to optimize its performance.

#### Mobile Technology for Community Health

MOTECH was launched in rural Ghana in 2010 in the Upper East region and later scaled up to seven districts across four regions [24]. The project aimed to leverage mHealth to increase the quantity and quality of prenatal and neonatal care in the Upper East region and create a replication in the Awatu Senya district and to improve health outcomes for mothers and their newborn babies [23]. MOTECH was scaled in clusters over a 3-year period to reach 78.7% (170/216) of Ghana’s districts [14].

The system has a component for maternal clients called Mobile Midwife app as well as the nurses’ apps [23]. The Mobile Midwife service provides pregnant women and their families with SMS text messages or voice messages that provide time-specific information about their pregnancy each week. These messages include alerts and reminders for care-seeking, actionable information and advice, and educational information. The messages were written in local languages.

Maternal clients can register for Mobile Midwife through either a community health worker who captures their details on a MOTECH registration form on the phone or by calling the MOTECH call center [23]. Users who do not have a personal or household phone may access their messages by calling a toll-free number from a phone on any telecommunications provider in the country. Once connected to MOTECH, the user interacts with the Mobile Midwife interactive voice response (IVR) system.

#### MomConnect

The MomConnect initiative is run by the country’s department of health [29]. The initiative sends SMS messages to maternal clients and new mothers in South Africa. “In three years, MomConnect has been taken to scale to reach over 95% of public health facilities and has reached 63% of all pregnant women attending their first antenatal appointment” [29]. MomConnect provides maternal clients with maternal health information and encourages them to register at an antenatal care clinic. It is expected that the intervention would provide a valuable service to new mothers, complementing the current set of health care services by informing mothers about maternal health and childcare [26]. Maternal clients subscribe to MomConnect via Unstructured Supplementary Service Data (USSD). To register on the system, a nurse must first confirm that the woman is pregnant [29].

SMS text messages sent to the maternal clients include antenatal care and access to care during labor, diet and nutrition, nonpregnancy-related infections, hypertension, newborn care, breastfeeding, and immunization. The system sends between 1 and 3 messages per week, depending on the stage of the pregnancy. The messages continue until the child is 1 year old [26]. The registration and the sending and receiving of messages are free of charge to the user. If a mother does not own a phone, she can opt to receive the messages via a phone owned by an acquaintance [26]. Maternal clients can register for the MomConnect service at any public health clinic in the country. MomConnect also has a help desk where mothers send messages. The messages are forwarded to the management of the concerned health facilities [29].

#### Chipatala Cha Pa Foni

CCPF (translates to *Health Center by Phone*) is a health hotline that was started in one district in Malawi in 2011. The initiative was later scaled up to the entire country, available 24 hours every day [35]. It was started as a pilot in the Balaka district, which was experiencing a high maternal mortality rate [38]. During the pilot phase, the intervention provided only maternal and child health services. The topics of calls ranged from danger signs needing emergency care to maternal clients calling to inquire about their expected due date [38]. Callers were provided with one-on-one health counseling with a care provider and were encouraged to provide home-based care and to seek appropriate care for themselves or their children when appropriate.

Furthermore, maternal clients were registered for the tips and reminders service during their first call. This service provides women with the opportunity to receive text messages or listen to recorded messages through the IVR system about how to care for themselves and their infants [38]. Messages were targeted to provide relevant and timely health information and reminders based on the stage of pregnancy or age of the child, such as reminders for antenatal care visits; birth planning; immunization timing; and the promotion of positive health behaviors, such as mosquito net use and exclusively breastfeeding [38].

The intervention evolved to become a general hotline, and the IVR system expanded to include different topics (in addition to the pregnancy topic), such as nutrition and hygiene [35]. Anyone could access the IVR system to speak with a hotline care provider or listen to specific messages [35]. From June 2019, the CCPF has been fully owned by the Government of Malawi, Ministry of Health [35].

## Results

### Overview

Our findings suggest that maternal clients appropriate mHealth interventions regardless of their mobile ownership status. Using MTA, the findings of this review were synthesized using the stages of the appropriation process, namely, adoption, adaptation, and integration (appropriation). We identified a number of factors as enablers and hindrances at different stages of appropriation. Table 1 summarizes the findings.

This section discusses the factors that influenced the different phases of appropriation of maternal mHealth interventions.

**Table 1:** Summary of findings.

Stages of appropriation	Enablers	Hindrances
Level 1: adoption	Easy to use [23,26,27,31,35]; content in local languages [23,24,35]; able to access the intervention on any mobile phone [13,30,31,35]; use of methods familiar to users (eg, SMS) [13,23,26,29,33,36]; and clear messages [14,23,27,31-33]	Inconsistent network connection [23,24,28,31]; user timeouts [26,28]; mobile phone skills [31-33]; and low literacy levels [23,31-33,35]
Level 2: adaptation	New information learned [23,27,29,31-33,35]; trusting of the message [23,26,27,31,35,36]; convenience of the service [23,27,31-33,36]; able to share information with husbands and friends [23,31,33,34]; and able to get situation-specific advice [23,31,33]	Mobile numbers cannot be changed [29]; messages not delivered [23,29,32,33]; malfunction of the keypad or mobile phone [31,33]; call congestion [23,35]; and bottlenecks in voice messages [14,23]
Level 3: integration	Empowered in decision-making [27,31,33,35,36]; improved number of antenatal visits [13,23,27,31,35]; improved food and medicine consumption [27,31,36]; place of delivery (health facility) [14,23,27,31-33,35]; exclusive breastfeeding [23,27,29,31]; improved number of vaccines [23,26,27,31]; and improved number of postnatal visits [23,26,27,31]	Messages not useful [27-29]; miscarriage [23,28]; stillbirth [23,28]; and baby loss [23,28]

### Adoption Stage

The design of all the 3 interventions took the context of a transitional country into account. This helped to increase the chances of adoption for a wide range of clients. The adoption of the 3 mHealth interventions was influenced by (1) the low cost of accessing the intervention, (2) the frugality of the design of the interventions, and (3) the inclusion of clients with no mobile phones. The services on all the 3 interventions were provided free of charge to the user. This reduced the chances of others being excluded from benefiting from the intervention based on their economic status. In transitional countries, women are more severely disadvantaged than men; hence, this is particularly useful because the interventions primarily targeted underserved communities that are burdened by economic hardships [39].

The interventions were frugal in that they were based on technologies that work on basic phones (eg, SMS text messaging, USSD, and voice) [23,26,27,31,35]. Although there is a growing number of mobile phones in Africa, most poor and rural women do not own smartphones [13]. Furthermore, in rural areas, mobile phone networks may not always support internet-based apps [33]. In such a context, technologically sophisticated interventions based on smartphones would serve little purpose. In addition to the ubiquity of the functionalities across phone types, the use of basic phone functionalities also ensured that the users were already familiar with such functionalities from their normal mobile phone use [13,23,26,29,33,36].

All the 3 interventions were designed to cater to both clients who owned and those who did not own a mobile phone. The interventions allowed those who did not own phones to use third-party phones [13,30,31,35]. The CCPF used community volunteers to provide maternal clients access to mobile phones. However, MomConnect and MOTECH allowed women to use mobile phones of husbands and friends. Hence, maternal clients could adopt the interventions regardless of their mobile phone ownership status. However, for CCPF, the use of community volunteers faced a number of challenges, such as sustaining volunteer motivation, challenges in accessing volunteers, phone maintenance, and mobile phone charging [33].

CCPF and MOTECH had the option for the clients to call a hotline or to interact with the IVR system to retrieve voice messages [23,35,40]. Most maternal clients used the pushed (voice messages sent to the client’s mobile phone) or retrieved voice messages (voice messages that are listened on demand through the IVR system). Maternal clients interacted with the IVR system to access voice messages [23]. The preference for voice messages could be because of low literacy levels in rural areas, especially among women [31]. Furthermore, this could be due to the fact that some African communities are oral societies and, therefore, prefer voice messages over written text [31].

### Adaptation Stage

Adaptation occurred when a maternal client had registered for the intervention and had familiarized herself with the intervention. Adaptation was influenced by (1) the need to learn new information and practices, (2) convenience of the service, and (3) trustworthiness of the information. The new information that the maternal clients learned about maternal health and nutrition influenced appropriation. The CCPF baseline survey showed that clients could list the information that was new to them [32]. The clients may have valued the intervention as a source of new information because clients struggle to obtain information from the clinics, as the clinics are too busy and have long queues. Furthermore, because of the culture that limits women from talking to strangers about pregnancy-related matters, women might have shied away from seeking the information from face-to-face consultations with clinicians [40].

The maternal clients felt that the interventions were convenient for them [23,27,31-33,36]. When the client did not feel well during pregnancy, they called the call center to determine whether their condition required medical attention. They saved time and money by not traveling long distances to the health facility, only to be told that they did not require medical attention. In rural areas of transitional countries, maternal clients travel long distances to the nearest health facility, and raising transport costs are a challenge [31].

Maternal clients trusted the information they received from the interventions and trusted the call center workers [23,26,27,31,35,36]. All the interventions were part of the health services provided by the department of health of their respective countries, which could be the reason why the maternal clients trusted the information [13,35].

Furthermore, there is evidence that the clients used the interventions and the information provided by the interventions [23,27,29,31-33,35]. On the basis of the information obtained from the interventions, the clients could make decisions about seeking care [27,31,33,35,36]. The messages helped the maternal clients make better maternal and infant health decisions. The maternal clients felt empowered and felt they could manage their pregnancy [26].

### Integration Stage

Integration is reached when using mHealth interventions becomes routine in the maternal client’s everyday life. The integration of the intervention in the clients’ lives was influenced by (1) attitudes and behaviors of the user and (2) performance of the technology [32,41]. At this stage, the use of the mHealth intervention influenced the maternal clients to attend all antenatal care clinics, take medication and have a balanced diet, deliver at the health facility, take the child to the clinic, and receive all the vaccines. An independent evaluation of CCPF linked the intervention with improved knowledge of maternal and child health as well as certain behaviors, such as increased use of antenatal care clinics within the first trimester [13,23,27,31,35], increased use of a mosquito net during pregnancy and also for children under the age of 5 years [32,34], increased rates of early initialization of breastfeeding, and increased knowledge of health behaviors in pregnancy and the postnatal period [35]. However, the evaluation showed reduced use during the postnatal period [23,26,27,31]. This could be because of the fact that some clients found that the messages were not useful [40].

### Factors That Affect Appropriation

Appropriation of the intervention was affected in different ways at all stages. The factors may be categorized as personal and technological. Personal factors such as low levels of literacy [23,31-33,35] and low mobile phone skills [31-33] influence the likelihood of clients not adopting the intervention. For CCPF, nonadoption occurred because the majority of community volunteers and users were not familiar with the IVR system. One of the challenges that maternal clients encountered when using the IVR system was that the messages could not play [32]. This may have been caused by low mobile phone skills or malfunction of the system itself. This is similar to other findings, such as barriers to IVR use are related to lack of familiarity with the technology and social barriers, including lack of mobile phone use skills and infrastructure challenges [42]. The implementers of CCPF overcame this challenge by training community volunteers or community health workers who, in turn, trained the maternal clients in their communities [33]. Hence, when interventions are being introduced, there should be a provision of bespoke training to improve familiarity of the intervention among the communities [43].

The technical challenges were related to the actual phone [31,33] as well as the network [23,24,28,31]. One challenge was related to instances such as when a client loses a mobile number [29]. The client could no longer receive the messages because the system did not allow change of the mobile number. Messages sent to these numbers were recorded in the system as dropped. In some circumstances, because of the low quality of mobile phones, the keypad could not function properly for the clients to interact with the mHealth system or the mobile phone stopped working during the period in which the client was supposed to be using the mHealth intervention.

The challenge of unreliable networks and user timeout [26,28] hindered maternal clients from registering with the interventions. MomConnect clients used USSD to register. Although this function is ubiquitous across different mobile phone types, it is prone to both network and user timeouts. Mobile network providers place a high priority on voice calls; therefore, in areas where the service is limited, USSD sessions are dropped and replaced by voice calls [28]. This challenge during the registration into the interventions might demotivate potential clients from adopting the intervention. Furthermore, call congestion influenced the maternal client to not appropriate properly.

At the integration stage, unexpected circumstances forced some maternal clients to withdraw from the intervention. The most common reasons for withdrawing were miscarriages, stillborn babies, and baby deaths [23,28].

## Discussion

### Principal Findings

This study suggests that several enablers influence maternal clients appropriate maternal mHealth interventions. The interventions were available free of charge to the clients, were implemented on technologies that were familiar to the potential clients, and were enabled to use regardless of mobile phone ownership status. Furthermore, the study noted a myriad of factors that hinder maternal clients’ appropriation of technological interventions.

### Considerations of the mHealth Intervention Context

mHealth technologies are enablers in the provision of intervention services. The use of SMS text messaging ensured that mHealth implementers could reach the most vulnerable maternal clients in hard-to-reach areas. However, the same SMS technology has raised several challenges. In all 3 interventions, some pushed SMS messages (SMS sent by the intervention to the maternal client mobile phone) sent to maternal clients were dropped [28]. Several factors contributed to the dropped SMSs. Some SMSs dropped because the recipients’ mobile phones were off or unavailable. Users in rural areas with limited electricity infrastructure typically switch off their mobile phones to preserve battery power. However, unavailability was because of the poor coverage of mobile networks in rural areas. Furthermore, the delivery rate of the pushed SMS messages depended on the mobile service provider. The high SMS drop rate could also be explained by some policy about changing phone numbers. MomConnect did not allow their clients to change their mobile phone numbers, and the clients had to register their new numbers. As such, pushed SMS messages for clients who had lost their mobile phones were recorded as dropped [29]. Furthermore, the delivery rate of pushed messages was observed to be dependent on the infrastructure and network coverage of mobile service providers.

Owing to the oral culture and low levels of literacy among women in rural areas, voice messages could have been a more appropriate option for message delivery than SMSs. However, the findings show that the delivery rate for pushed voice messages for MOTECH and CCPF was lower than that for the pushed SMS text messages [24]. This points to the role of infrastructural limitations in the design of mHealth interventions. Although some technologies may be more appropriate than others based on context, the limitations in infrastructure do not always allow designers to adopt user-centric designs. These challenges allude to the trade-offs between the design goals for low-resource and underprivileged settings. For example, the goal of implementing frugal innovations may not be congruent with the goals of technical reliability. Although the use of USSD addressed the goal of providing a low-cost option that was ubiquitous across all types of phones, this option did not offer technical reliability. Similarly, the goal of the need for voice messages was incongruent with the goal of ease of use.

Potential candidates for exclusion were those who did not own mobile phones. All the 3 interventions sought to include maternal clients who did not have a mobile phone. All interventions included the option of using a third-party phone [23,33]. The provision of asynchronous messages afforded the clients who did not own phones the flexibility to negotiate mobile phone use with the phone owners. CCPF reported that approximately 20% of maternal clients who accessed the service used third-party mobile phones [35].

### The Influence of Usefulness on Appropriation

The usefulness of an mHealth intervention may enhance how maternal health clients appropriate it. In this study, the maternal health clients used the messages from the interventions to improve their knowledge on how to take care of themselves during pregnancy, how to prepare for birth, and how to care for the baby after birth. Our finding is similar to that of a study in Bangladesh, which noted that maternal clients found maternal health care information received from an mHealth intervention valuable [44].

The hotline for the CCPF afforded women an opportunity to ask questions and obtain advice from the hotline workers. This, to an extent, was a shift from the cultural practices of avoiding talking about pregnancy-related matters too early and with people outside one’s own family. The hotline consultation afforded the women a sense of anonymity; they could talk to a person who could not see them and, therefore, had no power to harm their pregnancy. Here, it can be argued that the intervention mediated the interaction between clients and health care providers. The literature also claims that this interaction has improved women’s freedom to talk about pregnancy with health care workers [45]. Furthermore, the hotline consultation allowed the women to talk about their pregnancy to a health care provider who was not from their community and who could not see them. Here, the women sought medical care while maintaining what was socially required of them.

### The Role of Community of Purpose in the Appropriation of Maternal mHealth Interventions

The findings showed that a *community of purpose* around the maternal client may be vital to the success of the mHealth intervention. A community of purpose is the voluntary coming together of individuals with commitments and an organization with a mission [12]. The community of purpose has different members who may have different roles but are working together toward a shared purpose. The main purpose of the maternal health community of purpose is to promote the well-being of maternal clients. The mHealth intervention was one of the tools that the community could use to achieve its goals. In all the interventions, a variety of stakeholders, such as community leaders, community health volunteers, nurses, traditional healers, and other key community members, were engaged in the design of the programs [23,35]. The involvement of these stakeholders in the design process ensured that the implemented interventions were contextually relevant and sensitive. Involving actors in the health sector and people within the communities helps to legitimize the information being disseminated by the intervention [23].

CCPF and MOTECH train communities to know how to support maternal clients at home and in their communities. For example, a community could arrange for the transport of maternal clients to the health facility on the onset of labor [46]. Communities of purpose support maternal clients by ensuring that the clients have access to the intervention, even in cases where they do not own a mobile phone [35]. Leaving out key stakeholders could have negative consequences on the appropriation of the intervention.

### Conclusions

This study analyzed how maternal clients appropriate mHealth interventions for maternal health. The study used the cases of three maternal mHealth interventions in sub-Saharan Africa. The study noted that a myriad of factors play a role in the way clients appropriate technological interventions at different stages of the appropriation process. The study also noted that the socioeconomic status of the intended clients may affect their appropriation. If the designers fail to take into account the context in which the intervention is deployed, the intervention may perpetuate and even exacerbate existing inequalities. Although mHealth interventions may serve to include maternal clients in the information society, there is always a risk that some people could be left behind if the mediating factors in the context are not considered. To reduce inequalities during the appropriation process, it is also recommended that the interventions seek to create and leverage on communities of purpose around the use of the intervention.

### Future Work

This study used secondary data to understand how maternal clients appropriate mHealth interventions. Future studies should consider using primary data. This study did not distinguish the appropriation based on mobile phone ownership. It is likely that maternal clients who do not own a mobile phone and use third-party access experience the appropriation differently. It would be interesting to explore how maternal clients who do not own mobile phones appropriate maternal mHealth interventions.

## Abbreviations

CCPF: Chipatala Cha Pa Foni
IVR: interactive voice response
MOTECH: mobile technology for community health
MTA: model of technology appropriation
USSD: Unstructured Supplementary Service Data
